# Elevation of *Pseudoskusea*, *Rusticoidus* and *Protomacleaya* to valid subgenera in the mosquito genus *Aedes* based on taxon naming criteria recently applied to other members of the Tribe Aedini (Diptera: Culicidae)

**DOI:** 10.1186/s13071-015-1247-x

**Published:** 2015-12-30

**Authors:** Richard C. Wilkerson, Yvonne-Marie Linton

**Affiliations:** Department of Entomology, National Museum of Natural History, Smithsonian Institution, Washington, DC USA; Walter Reed Biosystematics Unit, Museum Support Center, Smithsonian Institution, Suitland, Maryland USA; Department of Entomology, Walter Reed Army Institute of Research, Silver Spring, Maryland USA; Faculty of Preventative Medicine and Biometrics, Uniformed Services University of the Health Sciences, Bethesda, Maryland USA

**Keywords:** Tribe Aedini, *Aedes*, Subgenera, Elevation

## Abstract

**Background:**

*Pseudoskusea*, *Rusticoidus* and *Protomacleaya* were well-recognized, morphologically distinct subgenera within the genus *Aedes* prior to a series of taxonomic changes over the past 15 years by Reinert, Harbach and Kitching, when they were recognized as subgenera of the genus *Ochlerotatus.* In our recent effort to stabilize the Tribe Aedini, we synonymized these subgenera and associated species back into the genus *Aedes*, but incorrectly assigned them as putative informal groups, instead of reinstating them to subgenera.

**Conclusion:**

Here we formally elevate three traditionally recognized subgenera (*Pseudoskusea*, *Rusticoidus* and *Protomacleaya*) within the genus *Aedes*.

## Findings

The tribe Aedini is comprised of about a third of all recognized mosquito species, and includes many vectors of debilitating viral diseases to humans, such as Dengue and Chikungunya. Within the tribe, the genus *Aedes*, in the traditional sense, is the largest genus in the tribe with 932 species. Other aedine genera are *Armigeres*, *Eretmapodites*, *Haemagogus*, *Heizmannia*, *Opifex*, *Psorophora*, *Udaya* and *Verrallina*. During the past 11 years, based on a series of morphological phylogenetic studies by Reinert, Harbach & Kitching (RH&K) [1–4], and the taxonomic actions resulting from those studies, the original genus *Aedes* was split into 74 genera, reducing the genus *Aedes* from over 900 species [5–8], to only 12. Chief among the reasons given by RH&K to elevate so many genera was the author’s claim of an unreferenced “principle of equivalent rank.” This implied that if traditionally accepted genera were phylogenetically co-equal with other clusters of species in their analyses, the newly recognized groups should also be given similar taxonomic status. These taxonomic actions were highly controversial [9, 10] and resulted in wide-spread confusion about which names to apply to most vectors of disease organisms in genus *Aedes* (see Table one in [12]). For example, during this period, *Aedes japonicus* (Theobald), an invasive species and proven vector of West Nile virus and Cache Valley virus, was known variously as *Aedes* (*Finlaya*) *japonicus* [5], *Ochlerotatus* (*Finlaya*) *japonicus* [11], ‘*Ochlerotatus’* (‘*Finlaya’*) *japonicus* [1] and *Hulecoeteomyia japonica* [2].

Close scrutiny of the RH&K phylogenetic results and a reanalysis of their dataset led Wilkerson et al*.* [12] to the conclusion that based on the evidence provided by RH&K the classification changes they promoted and that resulted in the split of the well-known genus *Aedes* into so many genera, were not warranted. *Aedes* was therefore reinstated [12], but to preserve their phylogenetic hypotheses the RH&K genera were reduced in rank to subgenera of *Aedes*. Any subgenera in the fragmented RH&K system were reduced to putative informal group status [12]. Rationalization for reinstatement of genus *Aedes* to include all “traditionally” accepted species was based on opinions promoting a conservative approach to classification change based on new phylogenetic analyses [13–15]. These opinions were comprehensively solidified by Vences et al. [16] who, in detail, discussed the relationship between nomenclatorial utility and phylogenetic accuracy. As a guide to determine the suitability for classification changes they proposed a number of Taxon Naming Criteria (TNCs). Appropriate TNCs were cited to reinstate the “traditional” species in genus *Aedes* [12]. Central to these arguments reinstating genus *Aedes*, while retaining other traditional aedine genera were: TNC 2, Clade Stability; TNC 3, Phenotypic Diagnosibility; TNC 8, Manageability; TNC 10, Nomenclatural Stability, and; TNC 11, Community Consensus. Since, to these authors [12], there was no compelling evidence warranting changing the classification of traditional diagnosable genera, the traditional genera in tribe Aedini should be retained until strong, multiple lines of evidence are produced showing the contrary.

Following our recent publication reinstating the genus *Aedes* [12], we revisited the above rationale and realized that three traditionally recognized *Aedes* subgenera (*Pseudoskusea*, *Rusticoidus* and *Protomacleaya*), recognized as subgenera by RH&K in their genus *Ochlerotatus*, were incorrectly synonymized as putative informal groups [12], when they should have been reinstated as *bona fide* subgenera of the genus *Aedes*. All are diagnosable, well-known traditional groupings and should be retained as such. Taxonomic information for each subgenus, including important references and component species are given in Appendix.

## Conclusion and formal taxonomic action

Here, we formally retrieve *Pseudoskusea*, *Rusticoidus* and *Protomacleaya* from synonymy within the *Aedes* subgenus *Ochlerotatus* [12], and elevate all three as subgenera of the genus *Aedes*.

## Appendix

Taxonomic Catalog Citation

### Subgenus *Pseudoskusea* Theobald 1907 (as genus) [17].

Type species: *Skusea multiplex* Theobald.

Subgenus Synonym *Caenocephalus* Taylor 1914 [18] (not *Caenocephalus* van der Wulp, 1898 [19])

*bancroftianus* Edwards, 1921

*culiciformis* (Theobald, 1905)

*multiplex* (Theobald, 1903)

*postspiraculosus* Dobrotworsky, 1961

### Important References:

Dobrotworsky 1961 (tax., bion.; Australia) [20]

Dobrotworsky 1965 (tax., key, bion.; Australia) [21]

Lee *et al.* 1984 (tax., key, distr., bion.; Australia) [22]

Reinert 2000 (to subg. of genus *Ochlerotatus*) [11]

Reinert 2002 (F gen.*) [23]

Reinert *et al.* 2006 (phyl., class.; to genus) [2]

Reinert *et al.* 2008 (to subg. of genus *Ochlerotatus*) [3]

Wilkerson *et al.* 2015 (phyl., class.; to syn. of subg. *Ochlerotatus* of genus *Aedes*) [12]

Herein: to subg. of genus *Aedes*

### Subgenus *Protomacleaya* Theobald 1907 (as genus) [17].

Type species: *Culex trisereatus* Say

*aitkeni* Schick, 1970

*alboapicus* Schick, 1970

*amabilis* Schick, 1970

*argyrothorax* Bonne-Wepster and Bonne, 1920

*berlini* Schick, 1970

*bertrami* Schick, 1970

*braziliensis* Gordon and Evans, 1922

*brelandi* Zavortink, 1972

*buenaventura* Schick, 1970

*burgeri* Zavortink, 1972

*campana* Schick, 1970

*casali* Schick, 1970

*chionotum* Zavortink, 1972

*daryi* Schick, 1970

*diazi* Schick, 1970

*gabriel* Schick, 1970

*galindoi* Schick, 1970

*hendersoni* Cockerell, 1918

*heteropus* Dyar, 1921

*homoeopus* Dyar, 1922

*idanus* Schick, 1970

*impostor* Schick, 1970

*insolitus* (Coquillett, 1906)

*knabi* (Coquillett, 1905)

*kompi* Vargas and Downs, 1950

*metoecopus* Dyar, 1925

*niveoscutum* Zavortink, 1972

*podographicus* Dyar and Knab, 1906

*sandrae* Zavortink, 1972

*schicki* Zavortink, 1972

*schroederi* Schick, 1970

*sumidero* Schick, 1970

*tehuantepec* Schick, 1970

*terrens* (Walker, 1856)

*thorntoni* Dyar and Knab, 1907

*triseriatus* (Say, 1823)

*vargasi* Schick, 1970

*zavortinki* Schick, 1970

*zoosophus* Dyar and Knab, 1917

### Important References:

Sourcouf & Gonzalez Rincones 1912 (as *Promacleaya*: emend.) [24]

Schick 1970 (keys, Terrens Group) [25]

Schick 1970 (keys, Terrens Group) [26]

Zavortink 1972 (tax.: resurrected from syn. with *Finlaya*) [27]

Reinert 2000 (to subg. of genus *Ochlerotatus*) [11]

Reinert *et al.* 2009 (as *'Ochlerotatus'* (*'Protomacleaya'*) *sensu auctorum*) [4]

Wilkerson *et al.* 2015 (to syn. of subg. *Ochlerotatus* of genus *Aedes*) [12]

Herein: to subg. of genus *Aedes*

### Subgenus *Rusticoidus* Shevchenko and Prudkina 1973 (as subg. of genus *Aedes*; M*, key) [28].

Type species: *Aedes refiki* Medschid

*albescens* Edwards, 1921

*bicristatus* Thurman and Winkler, 1950

*krymmontanus* Alekseev, 1989

*lepidonotus* Edwards, 1920

*provocans* (Walker, 1848)

*quasirusticus* Torres Canamares, 1951

*refiki* Medschid, 1928

*rusticus* (Rossi, 1790)

ssp. *subtrichurus* Martini, 1927

*subdiversus* Martini, 1926

### Important References:

Reinert 1999 (tax., review) [29]

Reinert 2000 (to subg. of genus *Ochlerotatus*) [11]

Reinert 2002 (F gen.*) [23]

Reinert *et al.* 2008 (phyl., class.) [3]

Reinert *et al.* 2009 (phyl., class.) [4]

Wilkerson *et al.* 2015 (to syn. of subg. *Ochlerotatus* of genus *Aedes*) [12]

Herein: to subg. of genus *Aedes*

Important taxonomic information and key references for the three *Aedes* subgenera treated herein (www.mosquitocatalog.org, 13 Sept. 2015); associated species are listed by subgenera. [Tax. = taxonomy, phyl. = phylogenetics, class. = classification, bion. = bionomics, distr. = distribution, subg. = subgenus, syn. = snynomy, * = all or part of life stage is illustrated, F = female, M = male, gen. = genitalia, emend. = emendation]
